# Tensile Strength Characterization of Alkaline-Treated and Untreated Banana Fibres Using Weibull Statistics

**DOI:** 10.3390/ma18214833

**Published:** 2025-10-22

**Authors:** Maryam Sodagar, Nassim Edouard Lagrou, Thomas Gries

**Affiliations:** Institut für Textiltechnik (ITA), RWTH Aachen University, 52074 Aachen, Germanythomas.gries@ita.rwth-aachen.de (T.G.)

**Keywords:** banana fibre, Weibull distribution, natural fibre

## Abstract

Banana fibres (BFs), derived from the pseudo-stems of Musa acuminata, represent a widely available agricultural residue with strong potential as an eco-friendly reinforcement in composite materials—particularly in bio-based epoxy or thermoplastic systems used in automotive interiors, packaging, and lightweight construction. However, their inherent variability presents challenges for consistent and reliable mechanical characterisation. This study investigates the effect of wood ash treatment, an eco-friendly alternative to conventional alkaline processing, on the tensile strength of single BFs. Fibres were treated in aqueous wood ash solutions at two pH levels (12.4 and 13.5) and soaking durations of 3 h and 24 h, and then tested according to ASTM C1557. At least 50 valid tensile tests per series were performed, and the results were analysed using a two-parameter Weibull distribution to quantify characteristic strength and variability, complemented by reliability analysis to assess survival probability. Untreated fibres exhibited low characteristic strength (396.6 MPa) and a Weibull modulus of 1.79, confirming significant scatter. Treated fibres showed marked improvements: the highest characteristic strength was achieved at pH 13.5 for 3 h (552.8 MPa, m = 3.17), while the greatest uniformity was observed at pH 13.5 for 24 h (m = 4.62). Reliability curves confirmed superior performance of treated fibres, with 75% survival strengths up to 373 MPa compared to 198 MPa for untreated. These findings demonstrate that wood ash treatment enhances both the strength and reliability of BFs for sustainable composite applications.

## 1. Introduction

The search for sustainable reinforcements in composite materials has intensified as industries seek to reduce reliance on synthetic fibres such as carbon and glass, whose production carries high energy demands and environmental costs. Natural fibres, including flax and hemp, have gained recognition for their renewability, low density, and relatively low carbon footprint. However, their large-scale cultivation needs resources and can compete with agricultural land for food production, prompting interest in fibres sourced from agricultural residues.

Banana fibres (BFs), derived from the pseudo-stems of banana plants, offer a compelling solution. After fruit harvesting, the pseudo-stem—constituting about 60% of the plant mass—is typically discarded, generating nearly 400 million tonnes of biomass waste globally each year [1,2]. Banana fibres contain 71.08% cellulose, 12.61% Hemicellulose, 7.67% lignin, have a low density (~1.28 g/cm^3^), and exhibit tensile strengths between 529 and 914 MPa, making them both strong and lightweight. Their full biodegradability further enhances their appeal for eco-friendly composite applications [3,4]. Their extraction from agricultural waste not only avoids competition with food production but also valorises a widely available residue.

Recent studies have further highlighted the potential of banana fibres in diverse applications. Pilien et al. investigated their use in geopolymer-based mortars and showed that optimised mix ratios enhanced performance [5]. Nasr, Bekraoui et al. explored extraction and utilisation strategies, emphasising their suitability as reinforcement in composites [6]. Complementing these findings, Badanayak et al. provided a comprehensive review on the extraction, characterisation, and surface modification of banana pseudo-stem fibres, underscoring the technological pathways to improve their structural and interfacial properties [7].

To optimise BF performance in composites, surface modification is often necessary to improve fibre–matrix adhesion [8,9,10,11]. Alkaline treatments using sodium hydroxide (NaOH) are widely used to remove hemicellulose, lignin, and surface impurities, increasing surface roughness and enhancing interfacial bonding [12]. Despite their effectiveness, such treatments raise environmental concerns due to the use of industrial chemicals. More sustainable alternatives have recently been explored, including the use of wood ash—a by-product of biomass combustion containing naturally occurring alkaline compounds. Studies have shown that wood ash can modify fibre surfaces and improve mechanical performance, but its effect on the intrinsic tensile properties of single fibres [13,14,15], particularly from a statistical perspective, has received limited attention.

Natural fibres are inherently characterised by a high variability in their mechanical properties, arising from both biological and processing-related factors. Differences in cultivation conditions, retting methods, and extraction techniques, as well as the specific location of a fibre within the plant stem, lead to variations in morphology and strength. At the microscale, features such as kink bands, lumen irregularities, and surface flaws further contribute to this heterogeneity [16,17]. As a result, reporting only average tensile strength values is inadequate for assessing fibre performance with reliability. To address this, statistical approaches are employed, with the Weibull distribution proving particularly effective for modelling the strength of brittle and quasi-brittle materials, including natural fibres [18,19,20]. Within this framework, the Weibull modulus (m) quantifies the variability of strength data, while the characteristic strength (σ_0_) defines the stress level at which 63.2% of fibres are expected to fail. Together, these parameters provide a rigorous basis for evaluating both the uniformity and reliability of natural fibres in engineering applications.

This study investigates the tensile strength distribution of single BFs, both untreated and treated with wood ash under different processing conditions, using Weibull statistical analysis. By comparing the Weibull modulus and characteristic strength for each condition, the work aims to quantify how sustainable treatment methods influence not only the magnitude of fibre strength but also its consistency and predictability. The findings will contribute to the design of environmentally responsible composite materials with improved and more reliable performance.

## 2. Materials and Methods

### 2.1. Banana Fibre Preparation

**Figure 1:**
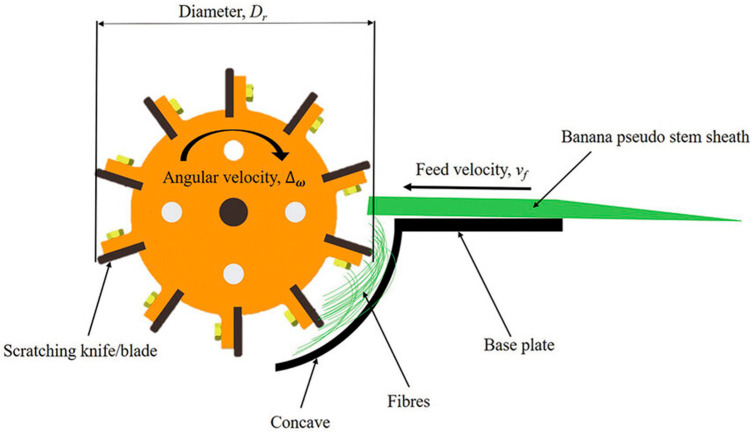
Schematic of banana fibre extracting machine [22].

BFs were sourced from Musa acuminata (*‘Dwarf Cavendish’*) cultivated in Mauritius. The fibres were extracted from the pseudo-stem sheaths using a banana fibre extraction machine (Riddhi Enterprise, Ahmedabad, India) installed at the University of Mauritius. Over time, various decortication machine designs with different processing capacities have been developed. These machines typically feature a rotating drum fitted with blades along its circumference, mounted on a shaft driven by an electric motor through a pulley system. The drum’s rotation provides the necessary beating and crushing action to separate fibres from the pulpy material [21]. A schematic of the fibre extraction process and test setup is provided in Figure 1. Following extraction, the fibres were subjected to a two-step drying process: first, air-dried for 24 h under tropical ambient conditions (23 ± 2 °C and 80 ± 5% relative humidity), and then oven-dried at 60 °C for an additional 24 h to reduce residual moisture content.

### 2.2. Wood Ash Treatment

Wood ash was obtained from a local wood-fired pizza outlet in Mauritius. The as-received ash was manually cleaned to remove unburnt wood fragments and large charcoal particles, followed by sieving to obtain a fine powder. The mesh size of sieve is not documented. Two alkaline solutions were prepared using tap water, with pH values adjusted to 12.4 (P1) and 13.5 (P2) by varying the quantity of wood ash added. Given the natural compositional variability of wood ash, the solutions were prepared to achieve the target pH rather than using a fixed mass ratio. The pH was measured using a Cyberscan pH meter (Eutech Instruments, Singapore).

The prepared solutions were left to stand for 24 h to allow sedimentation and then filtered through standard filter paper. BFs were immersed in the solutions at a fibre-to-liquor ratio of 1:30 and subjected to two soaking durations: 3 h (H3) and 24 h (H24). This produced four distinct treatment conditions combining the two pH levels and two immersion times.

The treatment conditions—specifically the pH levels of 12.4 and 13.5 and the durations of 3 and 24 h—were selected based on a balance between chemical effectiveness and practical feasibility. Lower pH values (around 12) mimic mild alkali treatment conditions reported in eco-friendly fibre modification studies, while a higher pH of 13.5 was chosen to explore the upper limit of the alkalinity achievable using wood ash, without introducing synthetic chemicals. These pH levels were deliberately chosen to investigate the effect of alkali concentration on fibre performance, as the extent of hemicellulose and lignin removal, and thus the mechanical enhancement, is known to be pH-dependent. Treatment durations of 3 and 24 h were selected to compare short-term surface modification with longer exposure that could promote deeper chemical penetration and structural changes, following established practices in NaOH-based fibre treatments.

Following immersion, fibres were neutralised by submerging them in tap water at a fibre-to-water ratio of 1:100, with a few drops of 80% acetic acid (approximately 0.5 mL per litre of water) added until the pH reached neutrality. The fibres remained in the neutralisation bath for 5 min before being rinsed thoroughly in fresh tap water. pH neutrality was confirmed using litmus paper. Treated fibres were then air-dried for 24 h, followed by oven drying at 60 °C for a further 24 h. The series codes, treatment pH values, and treatment durations are summarised in Table 1.

### 2.3. Single Fibre Tensile Test

Single fibre tensile tests were conducted in accordance with ASTM C1557 [23], using a Testometric M 500-50AT (The Testometric Company Ltd., Rochdale, UK) universal testing machine located in the Materials Engineering Laboratory at the University of Mauritius. The machine was fitted with a 10 kgf load cell and operated at a crosshead speed of 5 mm/min. Each fibre was mounted between pneumatically actuated, rubber-padded grips, with a gauge length set to 25.4 mm. A test was considered valid if fracture occurred within the gauge length, away from the grips, and within 30 s of loading. A minimum of 50 valid specimens was tested for each series to ensure statistical reliability.

To determine the tensile strength of each banana fibre, the ultimate breaking force was divided by the fibre’s cross-sectional area at the fracture location, following standard practice for single fibre tensile testing. Fracture regions were imaged using a Digimicro-Profi USB microscope, PCE Technology Co., Limited, Beijing, China, calibrated with a 100 µm scale graticule. Images were captured at 30× magnification under controlled lighting conditions. Assuming a circular cross-section, as supported by previous morphological analyses of banana fibres [15,24], the diameter was measured at four equidistant points around the fracture site using ImageJ software, National Institutes of Health (NIH), Bethesda, Maryland, USA. The average diameter was used to calculate the cross-sectional area. This systematic measurement procedure ensured that the dataset was both statistically robust and reflective of the intrinsic mechanical properties of the fibres.

### 2.4. Statistical Analysis of Fibre Strength Using the Weibull Distribution

In the present study, a two-parameter Weibull distribution was selected because it has been shown to provide more consistent and comparable estimates of natural fibre tensile properties than the three-parameter variant for short gauge length testing [25]. This model describes the probability density function, f(σi), and the cumulative distribution function, F(σi), as follows:

(1) $$f(\sigma_{i})=(\frac{\beta }{\eta }){\left(\frac{\sigma }{\eta }\right)}^{\beta -1}exp\left[-{\left(\frac{\sigma }{\eta }\right)}^{\beta }\right]$$

(2) $$F(\sigma_{i})=1-exp\left[-{\left(\frac{\sigma }{\eta }\right)}^{\beta }\right]$$

where β is the shape parameter (also referred to as the Weibull modulus) and η is the scale parameter (characteristic strength). The shape parameter reflects the variability of tensile strength within the fibre population: a low β value indicates high scatter and the presence of numerous defects, while a higher value implies greater uniformity. The scale parameter represents the tensile strength at which 63.2% of fibres are expected to fail [17,26], and is therefore a measure of the intrinsic load-bearing capability of the material.

To determine these parameters, tensile strength data were ranked in ascending order, and the median rank regression method was applied to generate a Weibull probability plot. The median rank position, $MR\left(i\right)$, for each data point was calculated according to:

(3) $$MR\left(i\right)=\frac{i-0.3}{N+0.4}$$

where i is the rank order of the tensile strength value and N is the total number of tested fibres. This transformation linearises the Weibull cumulative distribution function, enabling parameter estimation Via least-squares regression. In the resulting plot, the slope corresponds to the shape parameter β, while the intercept allows calculation of the scale parameter η. This methodology, described in detail by Torres et al. [27,28] has been adapted here to evaluate the tensile strength variability of untreated and wood ash-treated banana fibres.

While the Weibull probability plot is effective for determining the characteristic strength and variability of fibre tensile properties, it does not directly convey the likelihood that a fibre will survive a given applied stress. To provide an application-oriented interpretation of the results, reliability analysis was performed to estimate the survival probability of fibres as a function of tensile stress.

In this context, reliability R is defined as the probability that a fibre will not fail when subjected to an applied stress σ. For the two-parameter Weibull distribution, the reliability function is expressed as [29]:

(4) $$R\left(\sigma_{i}\right)=1-F\left(\sigma_{i}\right)=exp[-{\left(\frac{\sigma }{\eta }\right)}^{\beta }]$$

The reliability curve therefore represents the expected survival probability across the entire range of observed fibre strengths.

From the experimentally determined Weibull parameters, R(σ) was calculated for each series and plotted against tensile strength. This representation allows direct comparison of treatments at specific reliability thresholds (e.g., 75% or 50%), offering insight into performance across different regions of the strength distribution. Higher curves at a given reliability level indicate fibres with greater resistance to failure, while steeper slopes reflect greater uniformity in strength.

By combining this statistical approach with a large number of valid tensile tests per series (minimum 50), the present study ensures that both the average performance and the reliability of the fibres are accurately represented, providing a robust basis for comparing the effects of different treatment conditions.

## 3. Results and Discussion

**Figure 2:**
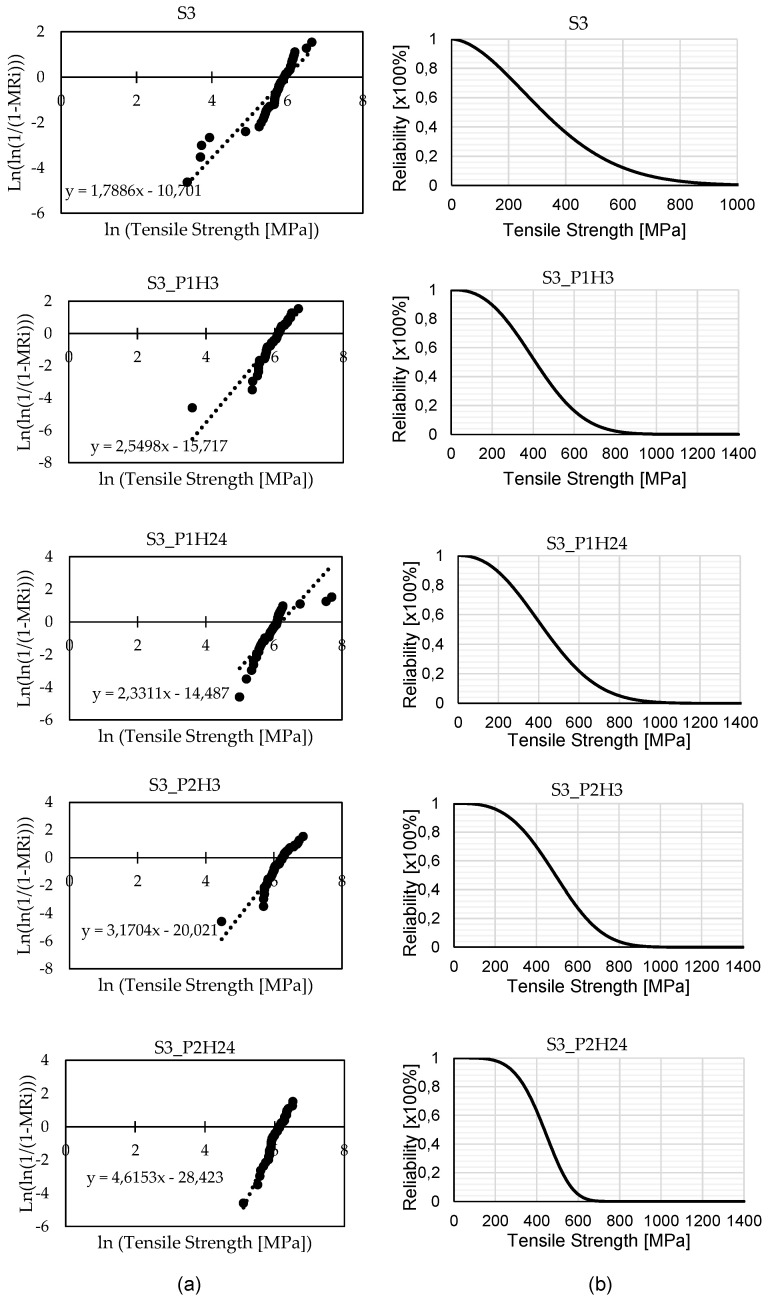
(**a**) Weibull probability plots for untreated and treated banana fibres, showing regression lines used to determine modulus and characteristic strength. (**b**) Reliability curves derived from Weibull parameters, indicating survival probabilities across applied stress levels.

The tensile strength distributions of untreated and wood ash-treated banana fibres were characterised using the two-parameter Weibull model. In the corresponding Weibull probability plot (Figure 2a), each black dot represents an individual experimental data point, while the dotted line denotes the linear regression fit based on the two-parameter Weibull equation, from which the Weibull modulus (*m*) and characteristic strength (σ_0_) were derived. The untreated fibre series (S) exhibited a Weibull modulus of 1.79 and a characteristic strength of 396.6 MPa, indicating a high degree of scatter in tensile strength and relatively low reliability. The low *m* value reflects the natural variability inherent to lignocellulosic fibres, which arises from microstructural heterogeneity, varying microfibril orientation, and the presence of flaws such as voids and surface defects.

All treated fibre series demonstrated both an increase in *m* and an enhancement in σ_0_ relative to the untreated fibres, indicating that wood ash treatment improves fibre uniformity and load-bearing capacity. Treatment at pH 12.4 for 3 h (S_P1H3) increased the Weibull modulus to 2.55 and the characteristic strength to 475.3 MPa. Extending the treatment duration at the same pH to 24 h (S_P1H24) resulted in m = 2.33 and σ_0_ = 500.0 MPa, suggesting a modest improvement in strength with a slight decrease in uniformity compared to the shorter-duration treatment. The most pronounced effect on tensile strength was observed for the pH 13.5, 3 h treatment (S_P2H3), which produced a characteristic strength of 552.8 MPa and a Weibull modulus of 3.17.

This outcome suggests that higher alkalinity over a moderate duration facilitates more complete removal of hemicellulose and surface impurities, which may enhance fibre–matrix interaction in composites, although this has not been directly confirmed Via SEM or FTIR in this study. In contrast, prolonged exposure to the same high pH for 24 h (S_P2H24) yielded the highest Weibull modulus of 4.62 but a reduced characteristic strength of 472.7 MPa. The increase in *m* indicates improved uniformity, possibly due to the elimination of weaker fibres from the population through degradation, while the reduction in σ_0_ points to partial damage to the cellulose structure under aggressive treatment conditions. This may result from excessive delignification of the fibre cell wall, which compromises cellulose integrity and thereby reduces load-bearing capacity.

The reliability curves (see Figure 2b) provide further insight by quantifying the tensile strength corresponding to a moderate-high survival probability of 75%, a level more representative of service expectations for natural fibre applications. At this reliability threshold, untreated fibres sustained 198 MPa before 25% of the population failed, reflecting their vulnerability to early failure events. All treated fibres exhibited substantial improvements. Treatments at pH 12.4 for 3 and 24 h (S_P1H3 and S_P1H24) achieved similar values of 292 MPa and 294 MPa, respectively, indicating that extending the treatment duration at this pH did not significantly affect the strength at this survival probability. The pH 13.5, 3 h treatment (S_P2H3) provided the highest 75% reliability strength of 373 MPa, with the pH 13.5, 24 h treatment (S_P2H24) slightly lower at 361 MPa. This trend parallels the characteristic strength data but also demonstrates that the benefits of higher alkalinity are maintained when considering a substantial proportion of the fibre population, rather than only the median performer.

When the Weibull and reliability analyses are considered together, the results indicate that both high-pH treatments deliver superior strength performance for the majority of fibres, while extended treatment at pH 13.5 offers slightly reduced strength but greater uniformity, as evidenced by its higher Weibull modulus. For applications where a balance between strength and predictability is important, such as semi-structural composite components, the pH 13.5, 3 h treatment appears optimal. Conversely, in contexts where dimensional stability and uniform performance are more critical than peak strength, the pH 13.5, 24 h treatment may be preferable. By integrating Weibull probability plots with reliability curves at an application-relevant survival probability, this study provides a more nuanced evaluation of treatment effects, ensuring that performance assessments align with the realistic reliability demands of natural fibre-based materials.

While the present study provides statistically robust conclusions, several limitations should be acknowledged. Firstly, the chemical composition of wood ash may vary depending on source material and combustion conditions, which could influence reproducibility. Secondly, only two pH levels and soaking durations were tested; intermediate or more granular conditions might yield further optimisation. Additionally, fibre morphology and surface chemistry were not investigated Via SEM or FTIR, which could provide deeper insights into treatment mechanisms. Finally, the study focused on single fibre testing, and the effect on composite performance remains to be evaluated.

In addition to the mechanical improvements observed, the practical and scalable use of banana fibres depends on the viability of their collection and processing. Banana pseudo-stems are an abundant agricultural residue generated after harvesting, particularly in tropical regions, and their utilisation does not compete with food production. Currently, fibre extraction is performed on a small scale using mechanical decorticators—such as those employed in this study—which are suitable for low-volume production and decentralised operations. However, if banana fibres find increased demand in industrial applications, such as composite industry, fibre production can be readily scaled by expanding extraction infrastructure and training programmes. Increased production would also create local employment opportunities, particularly in rural areas. While further development is needed to standardise quality and improve logistics, the basic supply chain—from post-harvest collection to fibre processing—is already technically feasible, making banana fibres a promising candidate for sustainable, scalable reinforcement in composite materials.

## 4. Conclusions

This study characterised the tensile strength distribution of untreated and wood ash-treated banana fibres using the two-parameter Weibull model, supported by reliability analysis. Untreated fibres exhibited low characteristic strength and high scatter, reflecting their intrinsic heterogeneity. Wood ash treatment improved both strength and reliability, with treatment severity influencing the balance between these properties. The pH 13.5, 3 h condition provided the highest characteristic strength, while the pH 13.5, 24 h condition delivered the greatest uniformity, albeit with a reduction in peak strength. Reliability curves at a 75% survival probability confirmed that all treated fibres outperformed the untreated fibres, with strength improvements approaching 90%.

Overall, the findings demonstrate that wood ash, a sustainable by-product, is an effective surface treatment for enhancing the performance of banana fibres. The choice of treatment condition should be guided by the application context: shorter-duration high-pH treatments are suited for maximising load-bearing capacity, whereas longer treatments are more appropriate where predictable behaviour is required. This work highlights the potential of eco-friendly treatments to advance the use of banana fibres in sustainable composite applications.

## Figures and Tables

**Table 1:** Annotation of series.

Series	Fibre	Treatment pH	Treatment Duration
**S**	Untreated BF	-	-
**S_P1H3**	Treated BF	pH of 12.4	3 h
**S_P2H3**	Treated BF	pH of 13.5	3 h
**S_P1H24**	Treated BF	pH of 12.4	24 h
**S_P2H24**	Treated BF	pH of 13.5	24 h

## Data Availability

The original contributions presented in this study are included in the article. Further inquiries can be directed to the corresponding author.
